# Supplementary material for: Key Genes of Lipid Metabolism and WNT-Signaling Are Downregulated in Subcutaneous Adipose Tissue with Moderate Weight Loss
Source: Nutrients. 2019 Mar 16;11(3):639. doi: 10.3390/nu11030639 (PMC6471921; doi:10.3390/nu11030639)
Supplement: Supplementary file 1 [file nutrients-11-00639-s001.zip › Supplementary Files/Schübel et al. Supplementary Materials.docx]

**Supplementary Figures**

269 assessed for eligibility

119 excluded:

97 not meeting inclusion criteria 22 declined to participate

2 withdrew

**49** allocated to **Intermittent calorie restriction**

**52** allocated to **Control**

Randomized (n=150)

**49** allocated to **Continuous calorie restriction**

3 withdrew

1 withdrew

**47** completed post-intervention

**46** completed post-intervention

**51** completed post-intervention

**Figure S1:** Participation rates in the 12-week intervention phase of the HELENA Trial


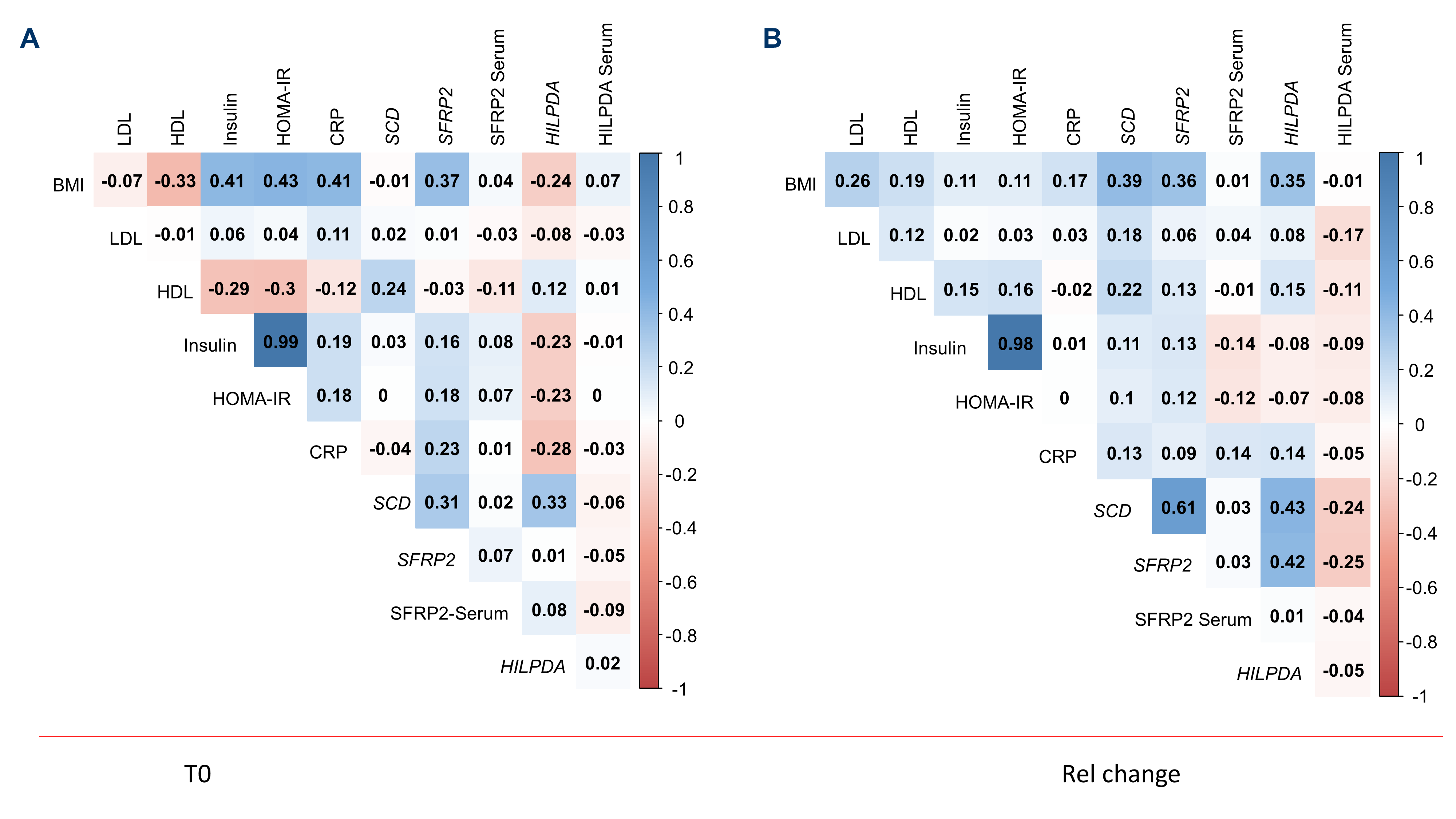


**Figure S2:** Cross-sectional correlations between *SCD*, *SFRP2,* and *HILPDA* microarray gene expression levels, serum levels of SFRP2 and HILPDA, BMI, and metabolic biomarkers (LDL-cholesterol, HDL‑cholesterol, insulin, HOMA-IR, CRP). **A:** Baseline data, and **B:** Log relative changes between baseline and week 12. Data are shown as Spearman coefficients adjusted for age and sex.


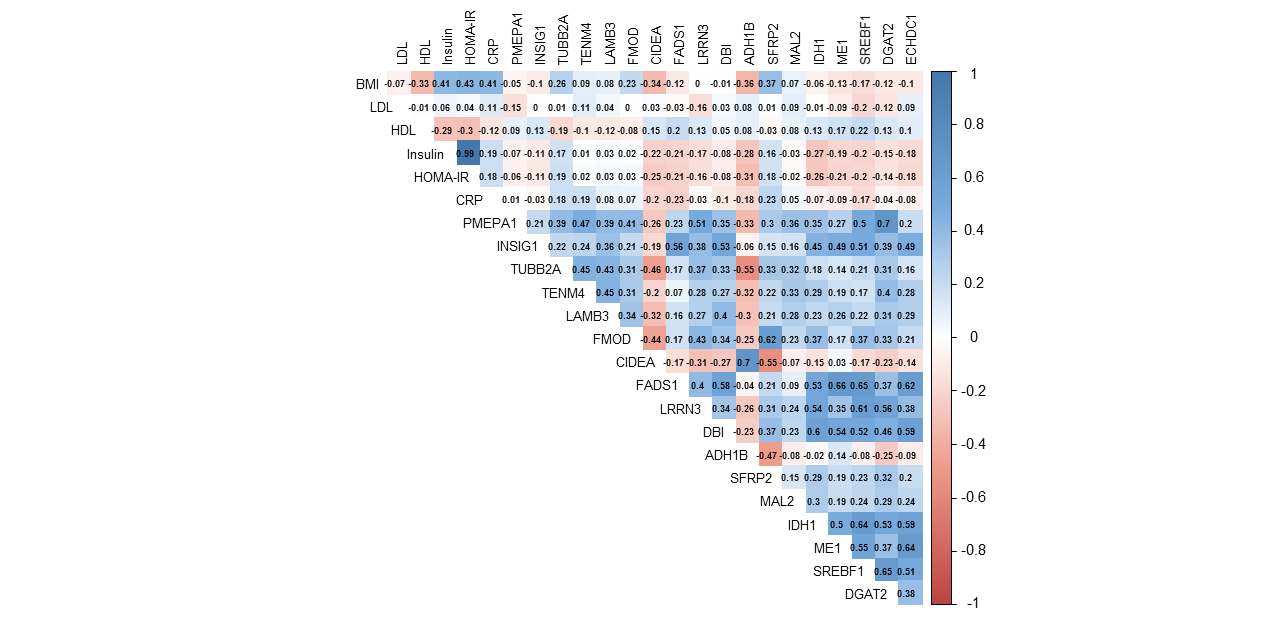


**
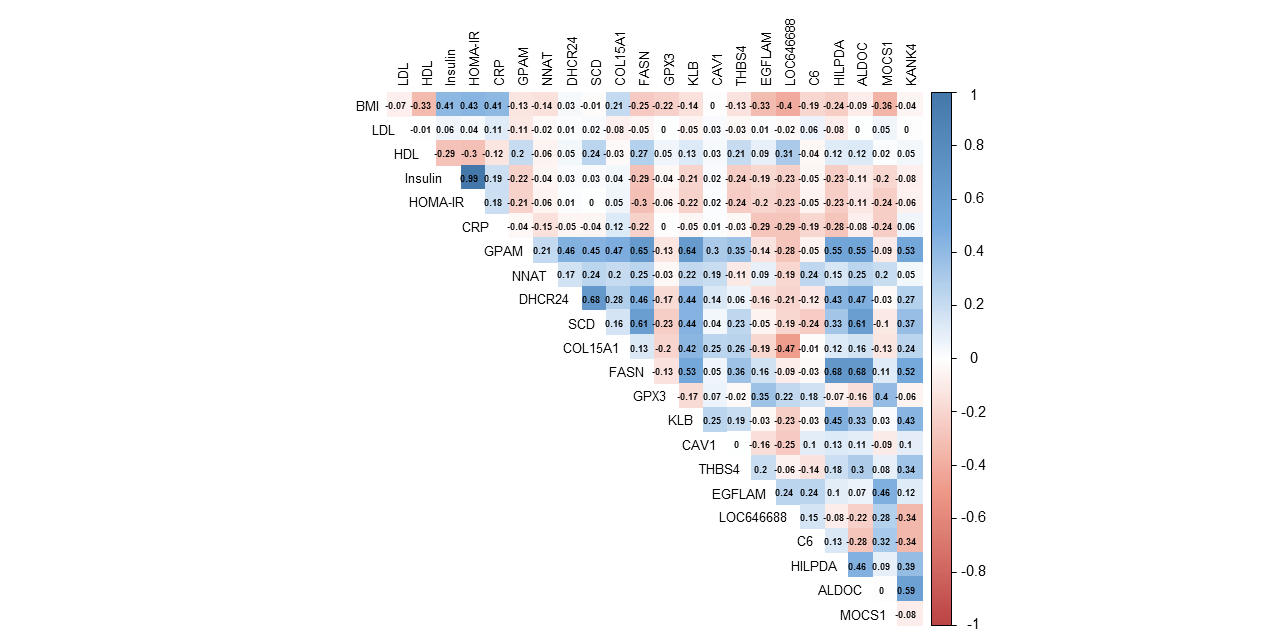
**

**Figure S3:** Cross-sectional correlations between microarray gene expression levels, BMI, and metabolic biomarkers (LDL-cholesterol, HDL-cholesterol, insulin, HOMA-IR, CRP) at baseline. Data are shown as Spearman coefficients adjusted for age and sex.

**
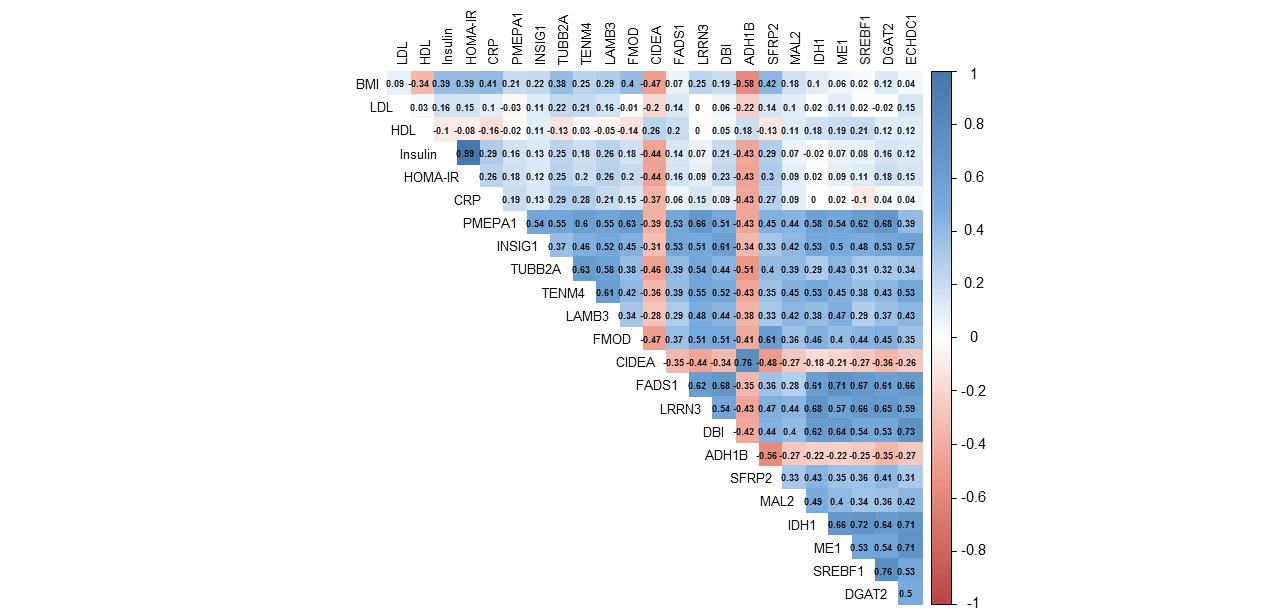
**

**
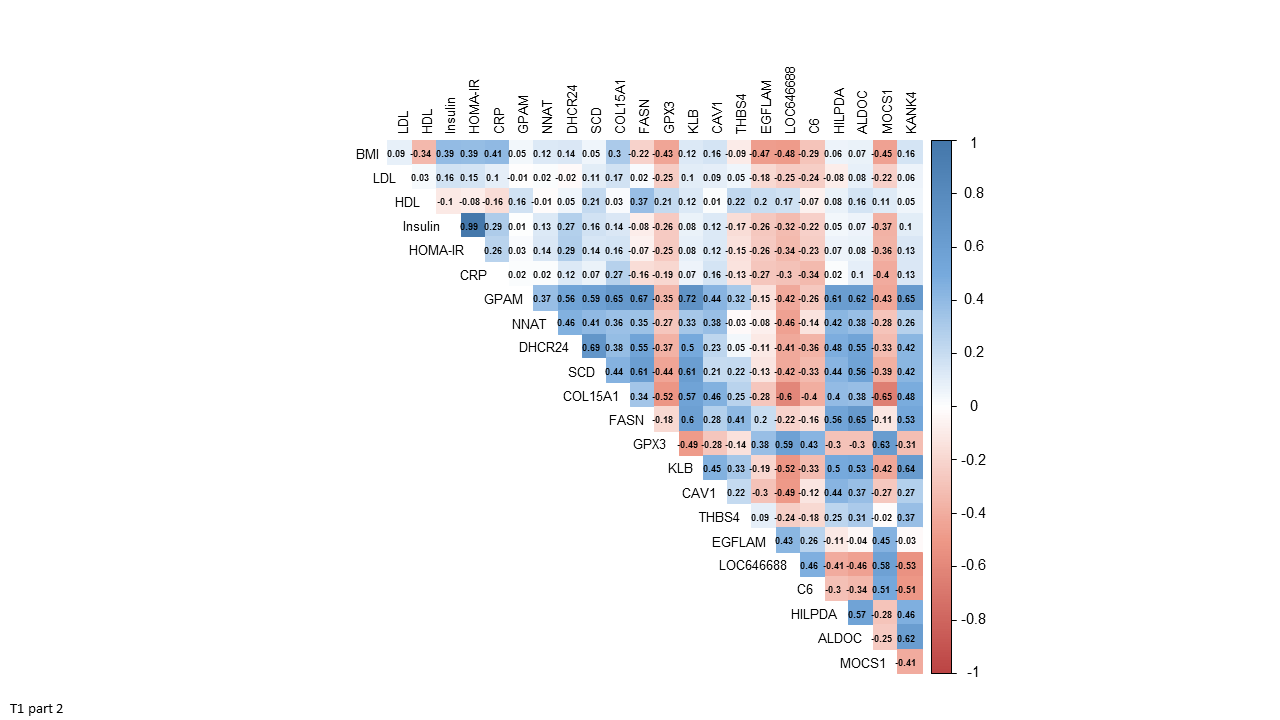
**

**Figure S4:** Cross-sectional correlations between microarray gene expression levels, BMI, and metabolic biomarkers (LDL-cholesterol, HDL-cholesterol, insulin, HOMA-IR, CRP) at week 12. Data are shown as Spearman coefficients adjusted for age and sex.


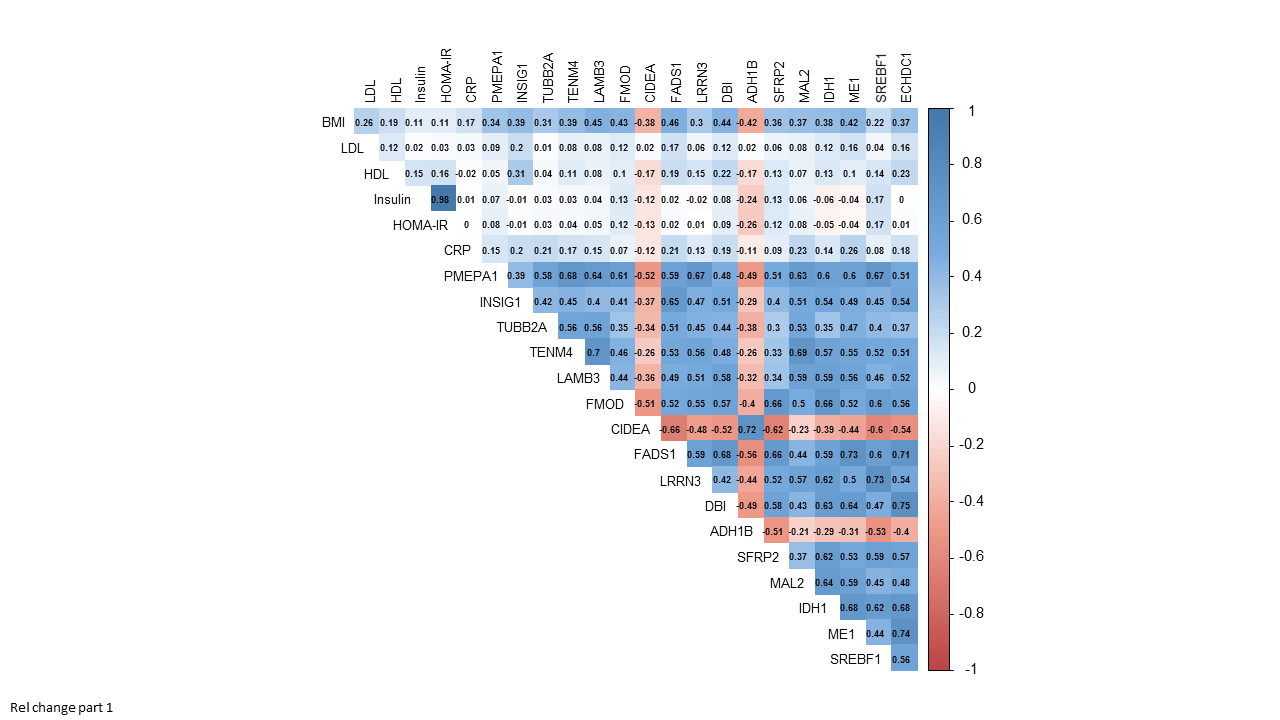

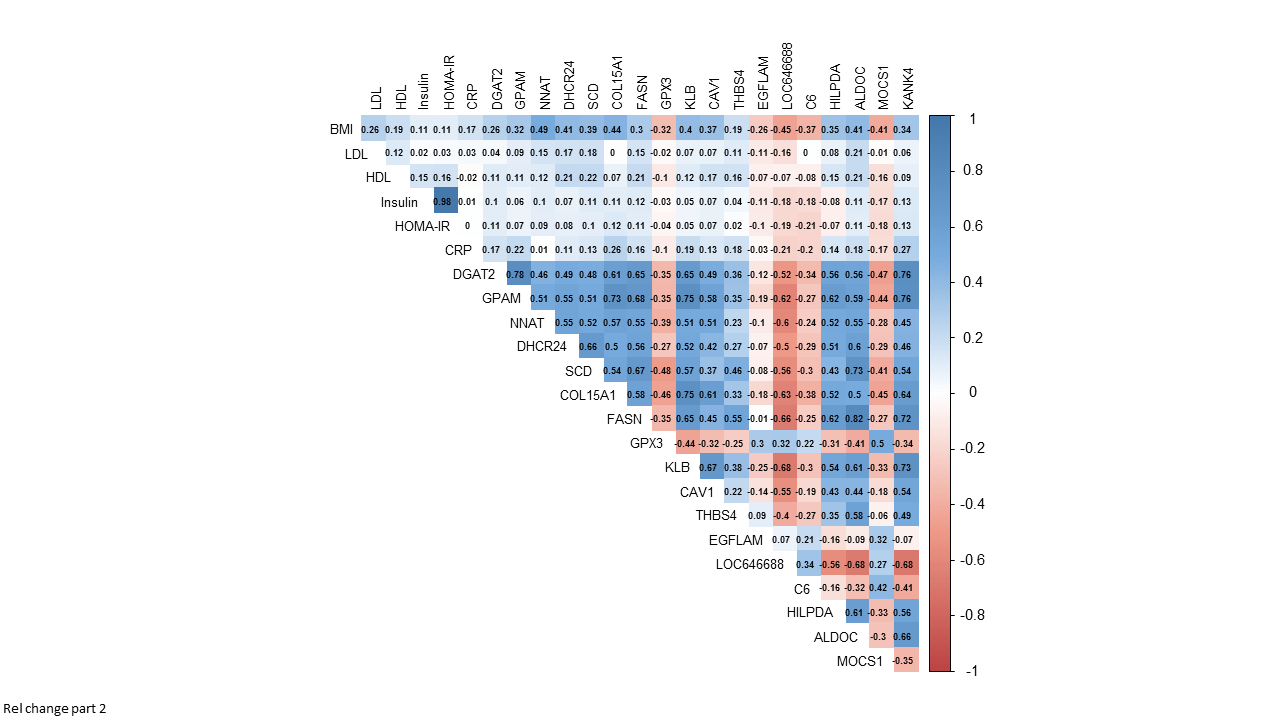


**Figure S5:** Cross-sectional correlations between microarray gene expression levels, BMI, and metabolic biomarkers (LDL-cholesterol, HDL-cholesterol, insulin, HOMA-IR, CRP) for log relative changes between baseline and week 12. Data are shown as Spearman coefficients adjusted for age and sex.

**Supplementary Tables**

**Table S1:** see Excel File: Literature review on diet- (SAT samples) and bariatric surgery- (SAT and VAT samples) induced weight loss with microarray gene expression of adipose tissue.

**Table S2:** Top 50 enriched pathways between lowest (reference) and highest weight loss quartile.

| **GO_ID** | **Name** | **Genes per**  **pathway** | **Regulated**  **genes** | **Z-score^a^** | **P-value^a^** |
| --- | --- | --- | --- | --- | --- |
| ***Down-regulated pathways*** | |  |  |  |  |
| GO:0016021 | integral component of membrane | 10187 | 188 | -7.1 | <0.001 |
| GO:0005739 | mitochondrion | 2710 | 99 | -4.8 | <0.001 |
| GO:0005789 | endoplasmic reticulum membrane | 1607 | 77 | -4.8 | <0.001 |
| GO:0005886 | plasma membrane | 7850 | 140 | -4.7 | <0.001 |
| GO:0055114 | oxidation-reduction process | 1397 | 66 | -4.5 | <0.001 |
| GO:0005783 | endoplasmic reticulum | 2514 | 82 | -4.2 | <0.001 |
| GO:0016491 | oxidoreductase activity | 1111 | 56 | -4.0 | <0.001 |
| GO:0006629 | lipid metabolic process | 884 | 56 | -3.9 | <0.001 |
| GO:0008152 | metabolic process | 1022 | 35 | -3.6 | <0.001 |
| GO:0005743 | mitochondrial inner membrane | 704 | 31 | -3.4 | <0.001 |
| GO:0046982 | protein heterodimerization activity | 777 | 16 | -3.1 | <0.001 |
| GO:0006631 | fatty acid metabolic process | 253 | 24 | -3.0 | <0.001 |
| GO:0006810 | transport | 3422 | 59 | -3.0 | <0.001 |
| GO:0005887 | integral component of plasma membrane | 2458 | 43 | -2.9 | 0.001 |
| GO:0043231 | intracellular membrane-bounded organelle | 1268 | 39 | -2.9 | 0.001 |
| GO:0005794 | Golgi apparatus | 2140 | 48 | -2.8 | 0.001 |
| GO:0042802 | identical protein binding | 646 | 19 | -2.8 | 0.001 |
| GO:0003824 | catalytic activity | 1110 | 34 | -2.7 | 0.001 |
| GO:0043547 | positive regulation of GTPase activity | 1158 | 17 | -2.7 | 0.001 |
| GO:0043234 | protein complex | 753 | 33 | -2.7 | 0.002 |
| GO:0000139 | Golgi membrane | 1073 | 35 | -2.6 | 0.002 |
| GO:0019901 | protein kinase binding | 680 | 17 | -2.6 | 0.001 |
| GO:0005764 | lysosome | 575 | 11 | -2.5 | 0.001 |
| GO:0042995 | cell projection | 1342 | 17 | -2.5 | 0.002 |
| GO:0005741 | mitochondrial outer membrane | 293 | 14 | -2.5 | 0.002 |
| GO:0006633 | fatty acid biosynthetic process | 126 | 20 | -2.5 | 0.003 |
| GO:0035338 | long-chain fatty-acyl-CoA biosynthetic process | 80 | 13 | -2.5 | 0.002 |
| GO:0005198 | structural molecule activity | 432 | 13 | -2.4 | 0.002 |
| GO:0014070 | response to organic cyclic compound | 195 | 17 | -2.4 | 0.003 |
| ***Up-regulated pathways*** | |  |  |  |  |
| GO:0003676 | nucleic acid binding | 2727 | 26 | 5.3 | <0.001 |
| GO:0003735 | structural constituent of ribosome | 320 | 12 | 4.7 | <0.001 |
| GO:0005840 | ribosome | 373 | 11 | 4.4 | <0.001 |
| GO:0006412 | translation | 577 | 16 | 4.4 | <0.001 |
| GO:0030529 | intracellular ribonucleoprotein complex | 618 | 13 | 4.3 | <0.001 |
| GO:0006413 | translational initiation | 264 | 13 | 4.2 | <0.001 |
| GO:0006364 | rRNA processing | 367 | 10 | 4.1 | <0.001 |
| GO:0003723 | RNA binding | 1574 | 24 | 3.9 | <0.001 |
| GO:0003677 | DNA binding | 4125 | 40 | 3.7 | <0.001 |
| GO:0000184 | nuclear-transcribed mRNA catabolic process, nonsense-mediated decay | 205 | 12 | 3.4 | 0.001 |
| GO:0008270 | zinc ion binding | 2326 | 45 | 3.4 | <0.001 |
| GO:0005730 | nucleolus | 1664 | 40 | 3.3 | 0.001 |
| GO:0006355 | regulation of transcription, DNA-templated | 4843 | 54 | 3.3 | <0.001 |
| GO:0044822 | poly(A) RNA binding | 1947 | 50 | 3.3 | <0.001 |
| GO:0005634 | nucleus | 11494 | 188 | 3 | <0.001 |
| GO:0005654 | nucleoplasm | 5283 | 77 | 3 | 0.001 |
| GO:0006351 | transcription, DNA-templated | 4230 | 51 | 2.8 | 0.002 |
| GO:0046872 | metal ion binding | 6658 | 121 | 2.6 | 0.002 |
| GO:0004674 | protein serine/threonine kinase activity | 815 | 11 | 2.5 | 0.008 |
| GO:0045087 | innate immune response | 757 | 11 | 2.5 | 0.007 |
| GO:0004672 | protein kinase activity | 1137 | 13 | 2.4 | 0.011 |

^a^ Z-score and p-values for pathway enrichment between weight loss quartile 1 and 4 were calculated with the *piano* package in R.
